# Supplementary material for: Socio-Territorial Inequities in the French National Breast Cancer Screening Programme—A Cross-Sectional Multilevel Study
Source: Cancers (Basel). 2021 Aug 30;13(17):4374. doi: 10.3390/cancers13174374 (PMC8430540; doi:10.3390/cancers13174374)

cancers

MDPI

Socio-Territorial Inequities in the French National Breast Cancer Screening Programme—A Cross-Sectional Multilevel Study

Quentin Rollet, Élodie Guillaume, Ludivine Launay and Guy Launoy

Table S1. *Départements*, sample size and FNBCSP participation rates.

Départements	Sample size	Participation rate
01 – Ain	7,745	56.5%
02 – Aisne	7,837	46.2%
06 – Alpes-Maritimes	17,926	40.9%
14 – Calvados	10,357	57.4%
17 – Charente-Maritime	11,011	52.6%
19 – Corrèze	3,723	64.8%
21 – Côte-d'Or	6,844	61.0%
23 – Creuse	2,108	50.1%
24 – Dordogne	6,984	58.8%
25 – Doubs	7,112	62.5%
27 – Eure	8,445	59.0%
29 – Finistère	14,604	64.0%
37 – Indre-et-Loire	8,508	68.3%
38 – Isère	15,561	57.7%
39 – Jura	3,856	59.7%
47 – Lot-et-Garonne	5,492	62.7%
50 – Manche	7,488	62.2%
51 – Marne	7,963	60.3%
54 – Meurthe-et-Moselle	10,600	52.8%
56 – Morbihan	11,902	68.2%
57 – Moselle	15,427	48.8%
62 – Pas-de-Calais	20,053	54.0%
63 – Puy-de-Dôme	9,515	59.9%
64 – Pyrénées-Atlantiques	10,107	62.4%
66 – Pyrénées-Orientales	7,559	58.4%
69 – Rhône	24,570	49.9%
70 – Haute-Saône	3,627	60.3%
71 – Saône-et-Loire	8,326	65.2%
73 – Savoie	6,265	63.3%
76 – Seine-Maritime	18,196	59.6%
80 – Somme	7,955	57.4%
81 – Tarn	6,149	56.6%
82 – Tarn-et-Garonne	3,675	60.3%
84 – Vaucluse	8,753	50.2%
86 – Vienne	5,995	56.9%
87 – Haute-Vienne	6,262	60.4%
89 – Yonne	5,421	59.5%
90 – Territoire de Belfort	1,880	60.2%
91 – Essonne	16,401	40.8%
93 – Seine-Saint-Denis	19,324	40.8%

95 – Val-d’Oise	16,072	45.8%
Total	397,598	55.1%

Figure S1. Participation rates by *départements* by European Deprivation Index population’s quintiles.

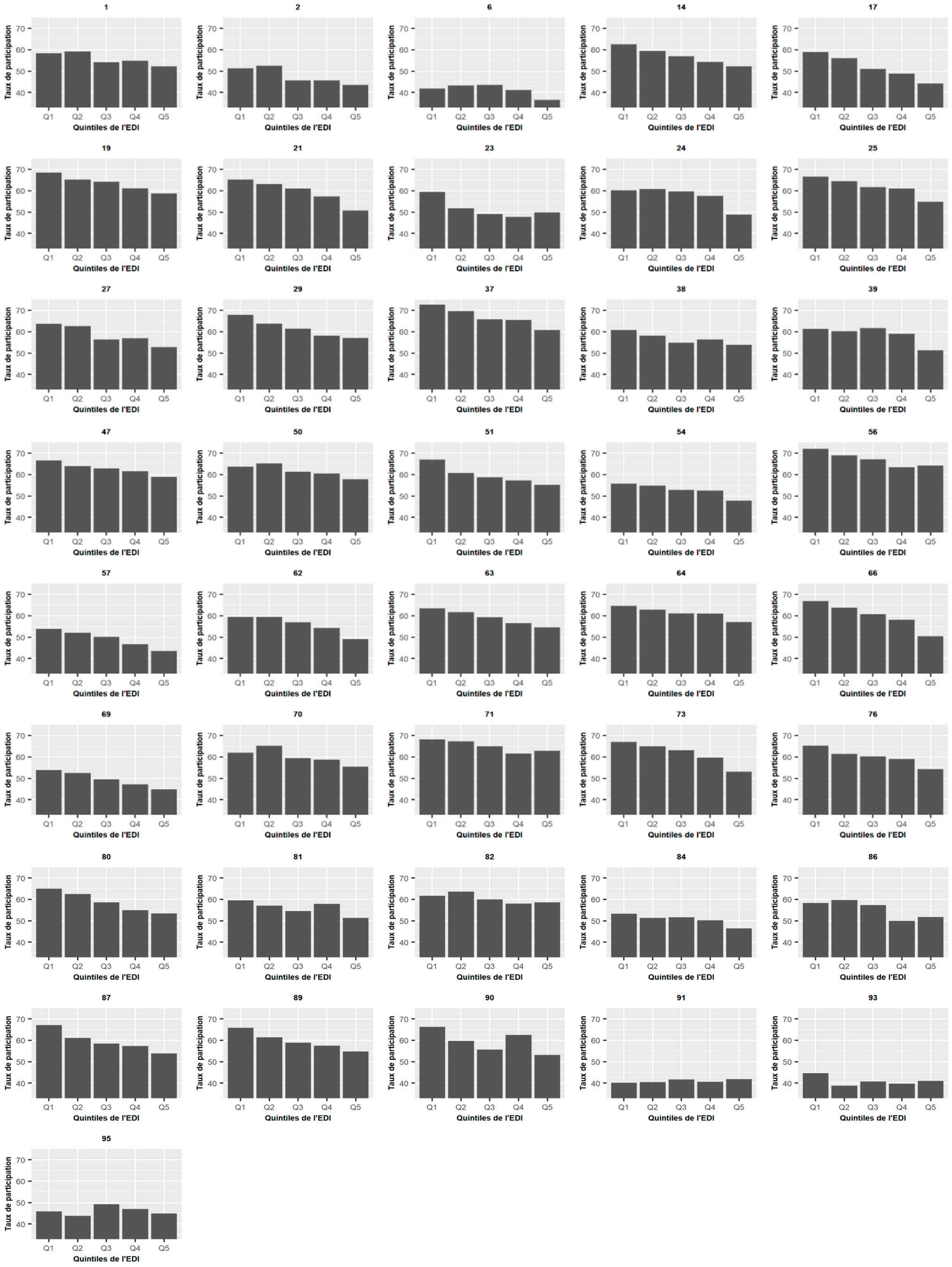

Figure S2. Participation rates by *départements* by travel time.

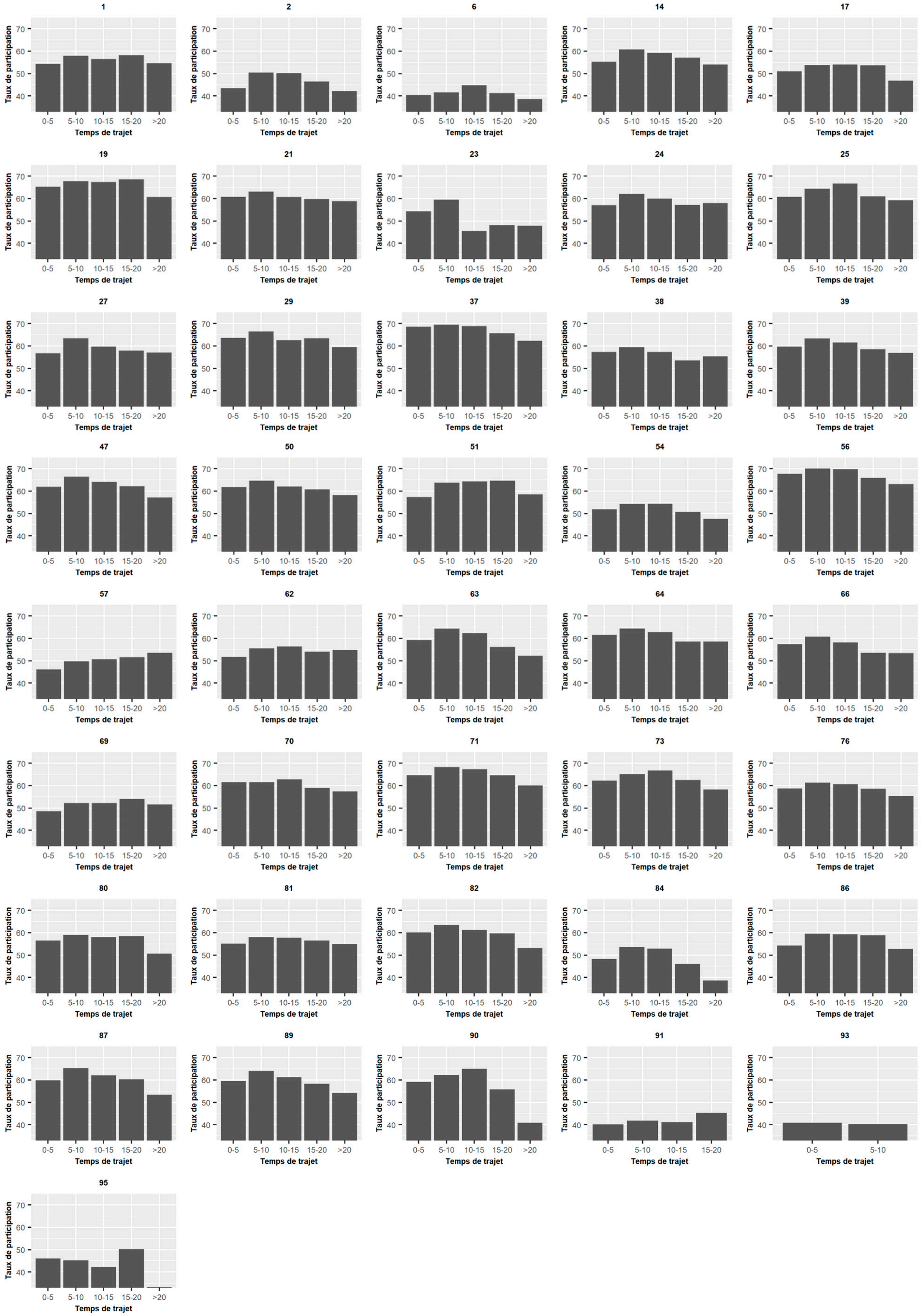

Supplement: Supplementary file 1 [file cancers-13-04374-s001.zip › cancers-1309393-supplementary.pdf]
